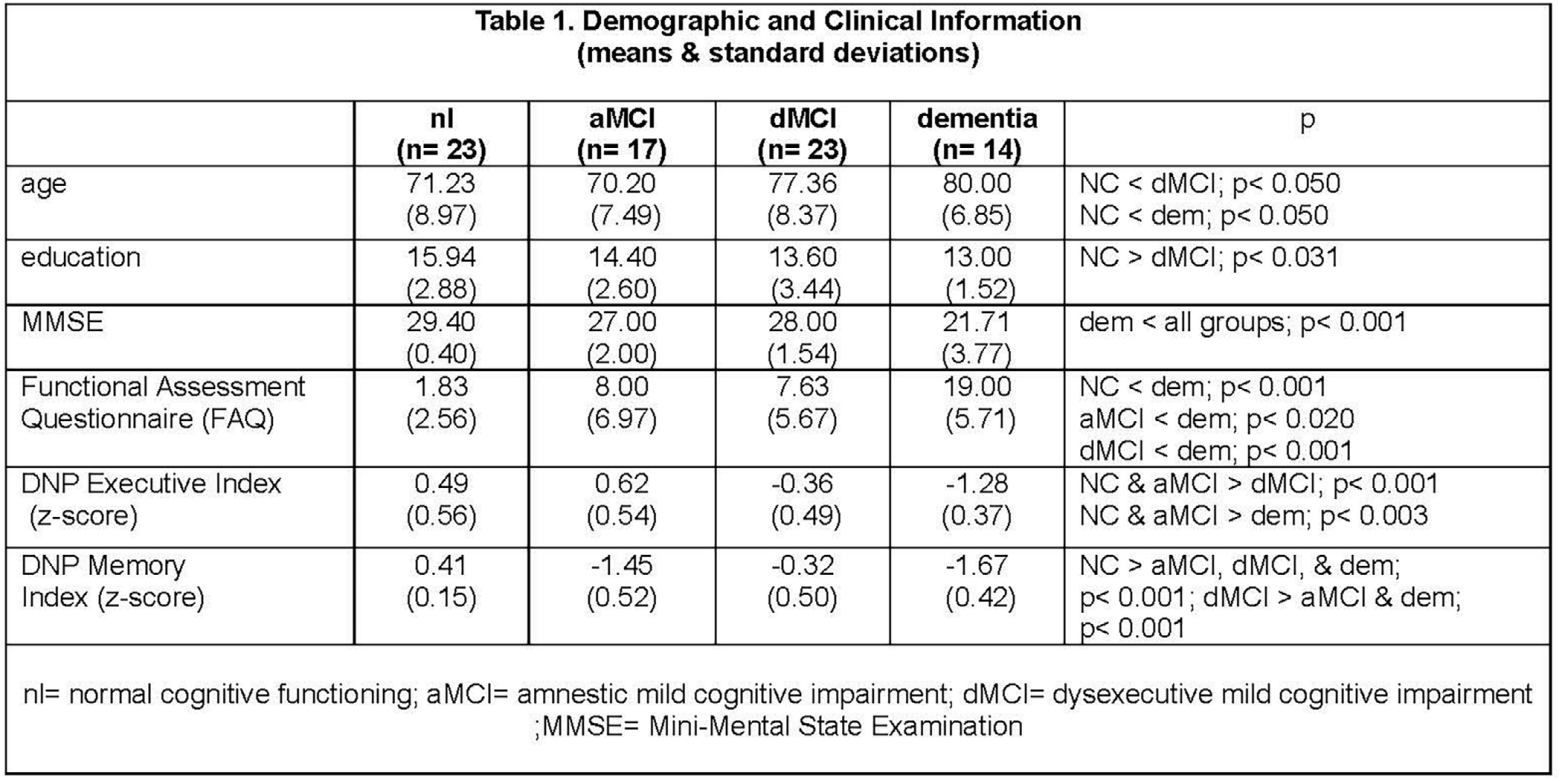# A Brief Digital Neuropsychological Protocol – I: Using Artificial Intelligence Assisted Technology to Assess Process and Errors

**DOI:** 10.1002/alz.094302

**Published:** 2025-01-09

**Authors:** David J. Libon, Rodney Swenson, Sean Tobyne, Catherine C. Price, Melissa Lamar, Stephanie Cosentino, Russell Banks, Ali Jannati, John Showalter, David Bates, Alvaro Pascual‐Leone

**Affiliations:** ^1^ Rowan University, Stratford, NJ USA; ^2^ University of North Dakota School of Medicine and Health Sciences, Grand Forks, ND USA; ^3^ Linus Health, Boston, MA USA; ^4^ University of Florida, Gainesville, FL USA; ^5^ Department of Psychiatry and Behavioral Sciences, Rush University Medical Center, Chicago, IL USA; ^6^ The Taub Institute for Research on Alzheimer’s Disease and the Aging Brain, Columbia University, New York, NY USA; ^7^ Harvard Medical School, Boston, MA USA; ^8^ Department of Neurology, Harvard Medical School, Boston, MA USA

## Abstract

**Background:**

There is an urgent need for neuropsychological screening tests that are easily deployed and reliable. We have developed a digital neuropsychological screening protocol that is administered on a tablet, automatically scored using artificial intelligence, and requires approximately 10 minutes to administer. This tablet‐administered protocol assesses the requisite neurocognitive constructs associated with emergent neurodegenerative illness

**Method:**

The digital protocol was administered to 77 ambulatory care/ memory clinic patients (Table1). The protocol is comprised of a 6‐word version of the Philadelphia (repeatable) Verbal Learning Test [P(r)VLT], three trials of 5 digits backward (BDST), and the ‘animal' fluency test. The protocol provides a panel of six traditional measures as would be obtained using paper/ pencil tests and manual scoring of (P[r]VLT free recall/ recognition hits, backward digit span, ‘animal' fluency output); a variety of outcome measures quantifying errors and the process used to bring tests to fruition; and two separate, norm‐referenced summary scores measuring executive control and memory.

**Result:**

Cluster analysis using the panel of 6 traditional measures classified participants into normal (nl = 23), amnestic MCI (aMCI = 17), dysexecutive MCI (dMCI = 23), and dementia (dementia = 23) groups. Subsequent analyses of error and process variables operationally defined key features associated with amnesia including rapid forgetting such as (P[r]VLT immediate free recall trial 2 vs. delay free recall (aMCI & dementia < dMCI & nl; p< 0.001), the production of extra‐list intrusion errors (dementia > nl; p< 0.002); profligate responding to recognition foils (aMCI & dementia > dMCI & nl; p< 0.001); key features underlying reduced executive measures (i.e., BDST perseveration/ related errors (dMCI & dementia > aMCI & nl, < 0.050); and the strength of semantic association from successive ‘animal' fluency responses (nl & dMCI > dementia; p< 0.028). The novel executive and memory index scores dissociated all four groups from each other (p< 0.014).

**Conclusion:**

This digitally administered and scored protocol yields patterns of impaired performance similar to paper/ pencil tests. The availability of both traditional and error/ process measures suggests that subtle, nuanced indications of early emergent illness may be identified in a fast, efficient, yet comprehensive way.